# CUX1—Transcriptional Master Regulator of Tumor Progression in Pancreatic Neuroendocrine Tumors

**DOI:** 10.3390/cancers12071957

**Published:** 2020-07-19

**Authors:** Sebastian Krug, Julia Weissbach, Annika Blank, Aurel Perren, Johannes Haybaeck, Volker Fendrich, Anja Rinke, Thomas Mathias Gress, Jonas Rosendahl, Patrick Michl

**Affiliations:** 1 Department of Internal Medicine I, Martin Luther University, 06120 Halle (Saale), Germany; sebastian.krug@uk-halle.de (S.K.); julia.weissbach@uk-halle.de (J.W.); jonas.rosendahl@uk-halle.de (J.R.); 2 Institute of Pathology, University of Bern, 3008 Bern, Switzerland; annika.blank@pathology.unibe.ch (A.B.); aurel.perren@pathology.unibe.ch (A.P.); 3 Department of Pathology, Neuropathology and Molecular Pathology, Medical University of Innsbruck, 6020 Innsbruck, Austria; johannes.haybaeck@i-med.ac.at; 4 Diagnostic & Research Institute of Pathology, Medical University of Graz, 8010 Graz, Austria; 5 Department of Endocrine Surgery, Schön Klinik Hamburg Eilbek, 22081 Hamburg, Germany; vfendrich@schoen-klinik.de; 6 Department of Gastroenterology and Endocrinology, Philipps-University, 35043 Marburg, Germany; sprengea@uni-marburg.de (A.R.); gress@uni-marburg.de (T.M.G.)

**Keywords:** neuroendocrine, pancreas, PanNET, chemotherapy, CUX1, apoptosis, recurrence

## Abstract

Recently, we identified the homeodomain transcription factor Cut homeobox 1 (CUX1) as mediator of tumour de-differentiation and metastatic behaviour in human insulinoma patients. In insulinomas, CUX1 enhanced tumour progression by stimulating proliferation and angiogenesis in vitro and in vivo. In patients with non-functional pancreatic neuroendocrine tumours (PanNET), however, the impact of CUX1 remains to be elucidated. Here, we analysed CUX1 expression in two large independent cohorts (*n* = 43 and *n* = 141 tissues) of non-functional treatment-naïve and pre-treated PanNET patients, as well as in the RIP1Tag2 mouse model of pancreatic neuroendocrine tumours. To further assess the functional role of CUX1, expression profiling of DNA damage-, proliferation- and apoptosis-associated genes was performed in CUX1-overexpressing Bon-1 cells. Validation of differentially regulated genes was performed in Bon-1 and QGP1 cells with knock-down and overexpression strategies. CUX1 expression assessed by a predefined immunoreactivity score (IRS) was significantly associated with shorter progression-free survival (PFS) of pre-treated PanNET patients (23 vs. 8 months; *p* = 0.005). In treatment-naïve patients, CUX1 was negatively correlated with grading and recurrence-free survival (mRFS of 39 versus 8 months; *p* = 0.022). In both groups, high CUX1 levels indicated a metastatic phenotype. Functionally, CUX1 upregulated expression of caspases and death associated protein kinase 1 (DAPK1), known as mediators of tumour progression and resistance to cytotoxic drugs. This was also confirmed in both cell lines and human tissues. In the RIP1Tag2 mouse model, CUX1 expression was associated with advanced tumour stage and resistance to apoptosis. In summary, we identified the transcription factor CUX1 as mediator of tumour progression in non-functional PanNET in vitro and in vivo, indicating that the CUX1-dependent signalling network is a promising target for future therapeutic intervention.

## 1. Introduction

Pancreatic neuroendocrine tumours (PanNET) represent a heterogeneous group of neoplasms with increasing incidence [1]. Only the minority of all pancreatic malignancies are of neuroendocrine origin [2,3]. However, the majority of PanNET patients are diagnosed with advanced metastatic disease [4,5]. Although we are able to stratify patients according to clinical, pathological and functional characteristics, the underlying tumour biology and the clinical course are still difficult to predict. Clinically, various prognostic factors have been described including age, Eastern Co-operative Oncology Group (ECOG) performance status, metastatic spread, tumour load, chromogranin A (CgA) levels and Ki-67 status [6,7,8,9,10]. Recently, two innovative tools have been developed to improve disease monitoring under systemic therapy: The tumour growth rate (TGR) has advantages over RECIST 1.1, including precise assessment of tumour growth and interpretation of therapy response [11]. The transcriptome-based NETest appears to be a robust and reproducible prognostic and predictive tool with superiority over single circulating biomarkers [12]. In PanNET undergoing cytotoxic chemotherapy, proliferation rate as initial criterion for treatment choice and response by imaging are most frequently used to guide treatment during the course of the disease [13,14]. The value of the MGMT promoter methylation status for the use of alkylating chemotherapeutics is still being debated which will be clarified by the ongoing clinical trial (NCT03217097) [15,16]. Alterations in DAXX and ATRX were found to be markers linked to poor prognosis and earlier recurrence, but are not yet routinely used for clinical decision making [17]. Reliable markers for the clinical routine are currently not available.

The transcription factor Cut homeobox 1 (CUX1), also called CCAAT-displacement protein (CDP) or Cut-like1 (CUTL1), is located on chromosomal band 7q22.1, and is expressed in multiple isoforms [18]. *CUX1* has been characterised as a haploinsufficient tumour suppressor gene, however, it is also overexpressed in many advanced cancers. Its role as initiator of tumour development and driver for cancer cell survival and tumour progression remains to be clarified. Several studies have proposed an important role for CUX1 in tumorigenesis and tumour progression in solid cancer [19]. Our group has shown that CUX1 modulates tumour progression in pancreatic adenocarcinoma by mediating tumour cell proliferation, migration and angiogenesis [20,21,22]. Furthermore, CUX1 expression is strongly associated with a less differentiated phenotype and short survival in patients with breast cancer [20], and is correlated with the malignant and metastatic phenotype of insulinomas [22].

The purpose of our study was to explore CUX1 in sporadic, non-functional, treatment-naïve and pretreated PanNET patients to investigate its impact on prognosis and to delineate underlying molecular mechanisms possibly leading to CUX1-induced therapy resistance. We identified a significant association of CUX1 expression with disease recurrence after curative resection, shorter PFS under cytotoxic therapy, metastatic spread in two independent patient collectives and as a potent regulator of the anti-apoptotic machinery, thereby serving as a new, promising therapeutic target.

## 2. Materials and Methods

### 2.1. Patients

Tissue-micro arrays (TMAs) of PanNET tissues from 104 patients were used for immunohistochemistry [17]. The use of this patient material was approved by the local ethics committee (Bern: number 15-04-12). Additionally, 93 consecutive patients with histologically confirmed PanNET who received chemotherapy were retrospectively identified from a database at the comprehensive cancer centre at the University Hospital of Marburg. Paraffin-embedded resected tissues of 31 patients from this cohort was available and analysed. The collection, storage and evaluation of patient-related information in our neuroendocrine tumour (NET) database was performed after informed consent was received, and with the approval of the local ethics committee at the University of Marburg (number: 105/12). This study was conducted in accordance with the Declaration of Helsinki.

### 2.2. Immunohistochemistry, Construction of Tissue-Micro-Array and Evaluation

In brief, sections of paraffin-embedded blocks or TMA were stained after antigen retrieval (microwave in antigen-unmasking solution, Vector Laboratories, Burlingame, CA, USA) with rabbit polyclonal anti-CUX1 (1:40–1:200), as described previously [21]. For death associated protein kinase 1 (DAPK1) staining, tissues were incubated with a monoclonal anti-DAPK1 antibody (1:150, Cell Signaling, Frankfurt am Main, Germany (#3008) 1:150). Caspase 3 and cleaved caspase 3 antibodies were purchased from Cell Signaling (working solution 1:100). The proportion of the positive cells (nuclear and/or cytoplasmic) and the tumour area was estimated in a percentage and divided into scores (<10%-1, 10–50%-2, 51–80%-3, >80%-4). The final score was determined as a product of the intensity of the staining and the proportion of positive cells (minimum 0, maximum 12) [23]. Grading according to the revised WHO classification of tumours of the digestive system 2010 was based on Ki-67 available from the pathology reports [24]. Tumours were graded using the proposed grading system of Rindi et al. [25,26]. The quantification of CUX1, caspases and DAPK1 expression scores was performed independently and in a blinded fashion by a pathologist with long-standing expertise in pancreatic endocrine tumours (J.H.). Non-neoplastic cells (lymphocytes and endothelial cells) served as an internal positive control in all tissue sections. The expression status of CUX1 was subsequently correlated with the clinical outcome of the patients.

### 2.3. Cell Lines

The human pancreatic neuroendocrine cell lines Bon-1 and QGP1 were provided from T. Gress, Philipps University Marburg, Germany. Bon-1 cells were cultured in DMEM F-12 (Gibco, Invitrogen Corp. Waltham, MA, USA). QGP1 cells were grown in RPMI (Gibco, Invitrogen Corp.). Both media were supplemented with 10% FCS (Capricorn, Ebsdorfergrund, Germany). All cells were cultured in a humidified atmosphere containing 5% CO_2_ at 37 °C. The cell number was analysed by counting the cells in a Neubauer chamber. The amphotropic packaging cell line LinX was maintained in DMEM, 10% foetal bovine serum, 250 mg/mL gentamicin and 100 mg/mL hygromycin B (Roth, Karlsruhe, Germany). Bon-1 cells stably expressing CUX1 were generated as previously described [22].

### 2.4. RNA Isolation, Cdna Synthesis and Real-Time PCR

RNA extraction was performed as previously described and according to the manufacturer’s instructions [22]. Sequence-specific primer pairs were designed using the Primer Express Software (Applied Biosystems, Waltham, MA, USA). RPLP0 was used as an internal standard (forward, 5′-GTC GGA GGA GTC GGA CGA G-3′ and reverse, 5′-GCC TTT ATT TCC TTG TTT TGC AAA-3′). Human cell-cycle-, apoptosis- and DNA damage pathway-focused gene expression profiling experiments comprising 84 genes each were performed using the RT2 Profiler PCR Array System (Qiagen, Hilden, Germany) according to the manufacturer’s instructions. Raw data of the profilers are available on request.

### 2.5. Immunoblotting

Cells were lysed in RIPA buffer supplemented with complete protease-inhibitor cocktail (Roche, Mannheim, Germany, 11697498001), phosphataseinhibitor mix (Serva, Heidelberg, Germany, 39050) and PMSF (Serva, 32395). Immunoblotting was performed as described previously [21]. Image J software was used for densitometry, according to the manufacturer’s instructions.

## 3. Statistical Design and Analysis

The comparison between clinico-pathological and laboratory features or immunohistochemistry was based on Fisher’s exact or Pearson’s correlation tests, as appropriate. Recurrence-free survival (RFS) was measured from date of surgery or biopsy to radiological diagnosis of recurrence. PFS was measured from the beginning of treatment to progression, death, or last follow-up. OS was measured from the beginning of treatment to the time of last follow-up or death. Survival was blotted by the method of Kaplan and Meier [27]. The statistical differences in RFS and PFS between groups of patients were estimated by the log-rank test [28]. Receiver operating characteristic (ROC) curve analysis was calculated to determine cut-offs in a binary system and to define sensitivity and specificity of a value. All statistical calculations were performed using SPSS (IBM SPSS Statistics). Differences were considered statistically significant when the *p* value was less than 0.05.

## 4. Results

### 4.1. CUX1 Expression in Treatment-Naive and Curatively Resected Pannet Patients

Previously, we identified the transcription factor CUX1 as mediator of tumour aggressiveness in insulinomas [22]. Based on our previous data in resected benign and malignant insulinomas we have established a histological scoring system for CUX1 using an immunoreactivity score (IRS) with a cut-off value of 8 determined by ROC analysis (AUC 0.74, *p* = 0.006, Figure S1A).

**Figure 1:**
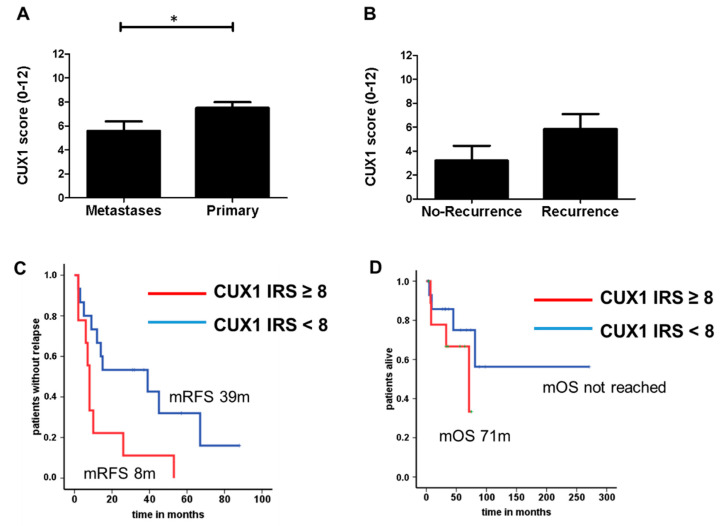
(**A**) Cut homeobox 1 (CUX1) immunoreactivity score (IRS) in patients with metastases (*n* = 40; mean: 5.6 ± 0.78 SEM) and primary tumours (*n* = 104; mean: 7.5 ± 0.48 SEM), * *p* = 0.038. (**B**) CUX1 IRS with recurrence (*n* = 19; mean: 5.8 ± 1.3 SEM) and no-recurrence (*n* = 5; mean: 3.2 ± 1.2 SEM) *p* = 0.31. (**C**) Log-rank test to assess mRFS depending on CUX1 expression, CUX1 IRS ≥ 8; *n* = 15, mRFS 39 months (95% CI 0 to 78.7 months); CUX1 IRS < 8; *n* = 9; mRFS 8 months (95% CI 6.6 to 9.4 months), *p* = 0.022. (**D**) Median overall survival (mOS) in CUX1 IRS ≥ 8; *n* = 16, mOS not reached; CUX1 IRS < 8; *n* = 10; mOS 71 months, *p* = 0.27.

Since no data are available on CUX1 expression in non-functional PanNET, we evaluated CUX1 expression in curatively resected non-functional PanNET patients (*n* = 104) without previous systemic therapy. For 37 of the 104 primary tissues, metastatic specimens (loco-regional lymph nodes) were also available. Clinicopathological features of the entire group are listed in suppl. file 1. CUX1 expression was almost undetectable in 21 primary tumours (20.2%) and 10 metastases (27.0%). The average staining intensity resulted in a higher CUX1 IRS in primary tumours, compared to metastases (7.5 ± 0.5 SEM vs. 5.6 ± 0.8 SEM, *p* = 0.038) (Figure 1A). Considering pathological and clinical characteristics of the present cohort, a strong CUX1 expression (IRS ≥ 8) was associated with a higher tumour grade (G1 vs. G2, *p* = 0.025, Fisher’s exact test), and there was a trend towards higher CUX1 positivity in primary tumours of patients with simultaneous distant metastasis (none vs. yes, *p* = 0.09, Fisher’s exact test) (Table S1). However, no significant correlation between the Ki-67 proliferation index (measured by Mib-1 antibody) and CUX1 staining could be. Since all patients underwent a surgical approach time to recurrence and disease-related death were the primary endpoints in this cohort. Patients who experienced recurrence during follow-up tended to have a higher CUX1 IRS compared to those without (3.2 ± 1.2 SEM vs. 5.8 ± 1.3 SEM, *p* > 0.05) (Figure 1B). When stratified by a CUX1 IRS cut-off 8, patients with CUX1 IRS > 8 had a significantly reduced median recurrence-free survival (RFS) of 8 months, compared to patients whose tumours showed an CUX1 IRS < 8 (Figure 1C), indicating that CUX1 protein expression is able to stratify patients into two distinct tumour-biologically relevant subgroups. This distribution also translated into overall survival, however, without reaching statistical significance (Figure 1D). While patients with recurrence and high CUX1 expression showed a median overall survival (mOS) of 71 months, the survival of patients with CUX1 low-expressing tumours did not reach the median (*p* = 0.27), indicating extended survival beyond the time of censoring due to end of follow-up.

### 4.2. CUX1 Expression in Pannet Patients Pre-Treated with Chemotherapy

**Figure 2:**
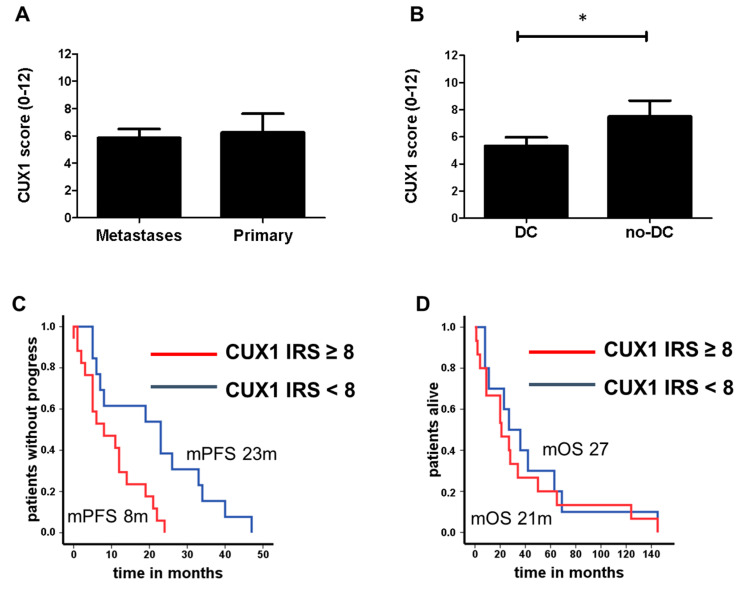
(**A**) CUX1 IRS in 43 PanNET tissues (35 metastases and 8 primary tumours) of 31 patients treated with chemotherapy (mean: 5.9 ± 0.6 SEM vs. 6.3 ± 1.4 SEM, *p* = 0.79). (**B**) CUX1 IRS in patients with disease control (DC, *n* = 31, mean: 5.3 ± 0.6 SEM) versus progressive disease (no-DC, *n* = 12, mean: 7.5 ± 1.2 SEM) * *p* = 0.045. (**C**) PFS in patients with predefined CUX1 IRS ≥ 8 (red line) compared to CUX1 IRS < 8 (blue line) (**D**) CUX1 IRS < 8 was 23 months (95% CI 5.8 to 40.2 months), CUX1 IRS ≥ 8 was 8 months (95% CI 0 to 16 months) (*p* = 0.005, figure) and OS (D, mOS 27 vs. 21 months, *p* = 58).

In addition to the cohort consisting of resected PanNETs described above, we investigated CUX1 expression in an independent cohort of non-functional PanNET with advanced, mainly non-resectable metastatic disease. From our study cohort of 93 PanNET patients treated with alkylating agents described previously [13] (for characteristics, see Table S2), 43 PanNET tissues of 31 patients were available for immunohistochemistry. Of these, 35 specimens (81%) were derived from metastases and eight specimens (19%) were primary tumours. Only two specimens (5%) were CUX1 negative (for representative images see Figure S2). No correlation between CUX1 immunoreactivity score (IRS) and tissue origin (primary tumour vs. metastasis) (5.8 ± 0.6 SEM vs. 6.3 ± 1.4 SEM, *p* = 0.8) was detectable (Figure 2A). There was also no association with the proliferation index in this group (Ki-67 ≥ 10%, AUC 0.55, sensitivity 54%, specificity 59%, *p* = 0.6). Interestingly, CUX1 IRS was significantly lower in patients with disease control (DC) under chemotherapy compared to progressive patients (no-DC) (5.3 ± 0.6 SEM vs. 7.5 ± 1.2 SEM, *p* = 0.045, Figure 2B). In line with this finding, we noted a significant inverse correlation between CUX1 immunoreactivity and progression-free survival. The median PFS in patients receiving chemotherapy with the predefined CUX1 IRS < 8 was 23 months (95% CI 5.8 to 40.2 months), as compared to 8 months (95% CI 0 to 16 months) for those with a CUX1 IRS ≥ 8 (*p* = 0.005, Figure 2C). In contrast, no significant differences were detectable with respect to overall survival (mOS 27 vs. 21 months, CUX1 IRS = 8.0, *p* = 0.58, Figure 2D). This suggests that CUX1 expression is not only prognostic, but might also predict response to chemotherapy. The fact that differences in PFS did not translate into differences in OS could be explained by subsequent therapy regimens and patients lost to follow-up. We examined possible associations between CUX1 expression and distinct clinicopathological features (Table S3). While CUX1 did not correlate with clinical or pathological parameters, such as age, sex or SMS-positivity, it was associated with more wide-spread extrahepatic and extra-lymphatic metastatic disease (e.g., bones, lung, peritoneal or spleen) (*p* = 0.021).

### 4.3. CUX1 Targets for Anti-Apoptotic Effects in Vitro

**Figure 3:**
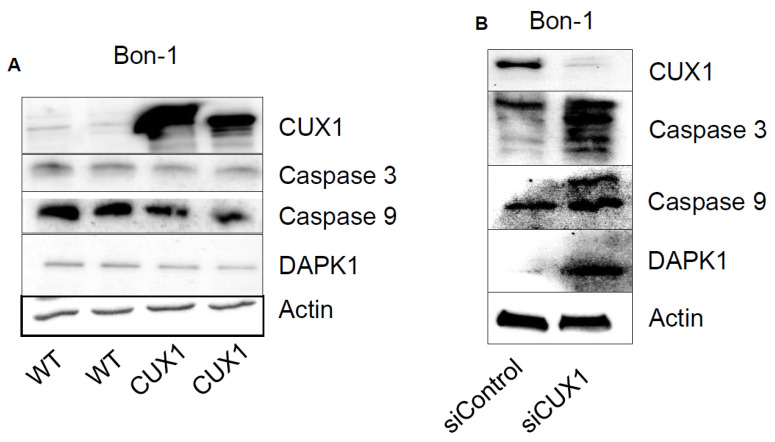
Regulation of caspase 3 and 9 and DAPK1 protein levels by Western blot analysis in Bon-1 cells (**A**). Two wildtype (WT) and two CUX1 overexpression clones were used. (**B**) CUX1 knock-down via siRNA in comparison to siControl in Bon-1 cells. Actin served as internal control.

To elucidate the transcriptional alterations underlying the CUX1-mediated impact on RFS and PFS in our patient cohorts, we examined potential CUX1-regulated targets in CUX1 overexpressing Bon-1 cells as an in vitro model by using an expression profiling approach focusing on genes involved in cell-cycle, apoptosis and DNA damage (Figure S3A–C and file S2). In line with its well-known role as transcriptional repressor, most differentially regulated genes were repressed by CUX1, particularly those involved in cell-cycle control and apoptosis signalling. Selected genes consistently regulated by CUX1 on mRNA level were subsequently validated on protein level by immunoblotting. Interestingly, caspase-3 and -9, as well as the DAPK1 (death-associated protein kinase 1) were repressed by CUX1 on protein level (Figure 3A). Consistent with this, we found an increased expression of caspase-3, -9 and DAPK1 upon siRNA-mediated depletion of CUX1 (Figure 3B). We aimed to confirm our findings by using the neuroendocrine tumour cell line QGP1. In contrast to Bon-1 (derived from a lymph node metastasis), QGP1 cells originate directly from a pancreatic neuroendocrine primary tumour [29]. In QCP1 cells, regulation of caspases-3 and -9 was readily confirmed, following the modulation of CUX1, whereas DAPK1 was not consistently affected (Figure S4A,B), confirming caspase-3 and -9 as CUX1 targets across cell lines.

### 4.4. CUX1 Targets for Anti-Apoptotic Effects in Vivo

**Figure 4:**
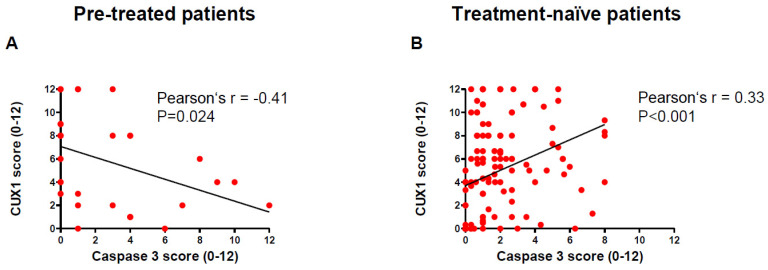
(**A**)Pearson’s correlation test between CUX1 und caspase 3 IRS in the pre-treated group. Number of pairs = 31, 95% CI -0.66 to -0.059, r = −0.41, *p* = 0.024. (**B**) Pearson’s correlation test between CUX1 und caspase 3 IRS in the treatment-naïve group. Number of pairs = 141, 95% CI 0.17 to 0.47, r = 0.33, *p* < 0.0001.

To validate CUX1 target genes in vivo, we assessed caspase-3 and DAPK1 in both PanNET cohorts, as mentioned above. In the group of PanNET patients pre-treated with cytotoxic chemotherapy, caspase-3 staining was rather low, with an average IRS of 2.7. 12 out of 31 specimens showing no caspase-3 staining (39%). Overall, there was a strong inverse correlation between CUX1 and caspase-3 (r = −0.41, *p* = 0.024) (Figure 4A). This is in line with the strong anti-apoptotic phenotype of CUX1 seen in many solid malignancies [18]. Interestingly and in contrast to pretreated patients, in treatment-naive patients CUX1 and caspase-3 had a positive correlation (r = 0.33, *p* < 0.001). Since the patient and tissue characteristics in this group was markedly different from the first cohort, with a majority of tissues being primary tumours and median caspase-3 IRS being lower than in the pretreated cohort, with an IRS of 1.8 in primary tumours and 2.0 in metastases, respectively. We hypothesise that a different stage and tumour biology, as well as sample origin between treatment-naïve and pretreated patients also impact on both CUX1 and caspases protein levels. In line with the in vitro findings, no robust correlation could be determined between CUX1 and DAPK1 in both human cohorts.

**Figure 5:**
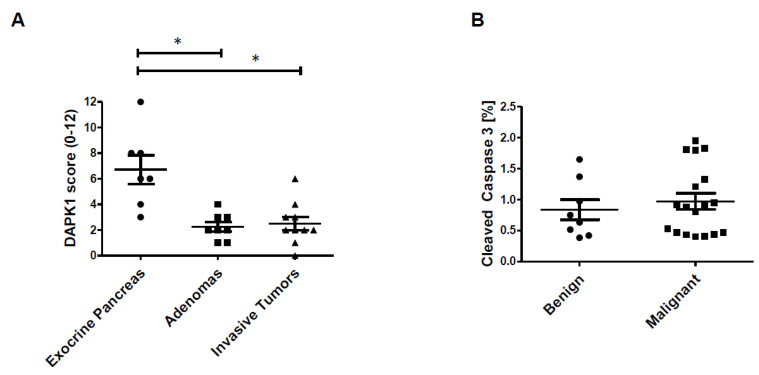
DAPK1 and caspase 3 IRS in different stages of tumorigenesis in the RIP1Tag2 mouse model. (**A**) Exocrine pancreas (*n* = 7, mean: 6.7 ± 1.1 SEM), adenomas (*n* = 8, mean: 2.3 ± 0.37 SEM) and invasive tumours (*n* = 10, mean: 2.5 ± 0.52 SEM). * *p* < 0.05. (**B**) Cleaved caspase 3 in benign (hyperplastic adenomas/angiogenic tumours, *n* = 8, mean: 0.84 ± 0.16) versus malignant (invasive tumours, *n* = 18, mean: 0.97 ± 0.13 SEM) tumours presented as positive cells in %.

We also investigated the pancreatic neuroendocrine tumour mouse model RIP1Tag2 for CUX1 regulated target genes. The immunohistochemistry of CUX1, DAPK1 and caspase-3 in benign (adenomas) and malignant lesions (invasive tumours) is depicted in Supplementary Figure S5. Previously, we already described a gradual increase in CUX1 RNA expression during tumour progression in the RIP1Tag2 model [22]. IHC analyses revealed reduced DAPK1 in endocrine cells compared to exocrine pancreas (IRS 2.25 vs. 6.71, *p* = 0.0003) (Figure 5A). There was no expression difference between adenomas and invasive tumours (IRS 2.25 vs. 2.5, *p* = 0.71). An increased staining intensity could be shown for total caspase-3, whereas cleaved caspase-3 was not significantly different during tumour progression (% of cleaved caspase-3 positive cells in adenomas and invasive tumours: 0.84 vs. 0.97, *p* = 0.55) (Figure 5B). These data indicate the described negative correlation of CUX1 and caspase-3 confirming the hypothesis of an anti-apoptotic path mediated by CUX1.

## 5. Discussion

Patients with metastatic PanNET show a median overall survival of approximately 6.5 years, which that strongly depends on the available therapeutic options, including surgery and systemic approaches [5,10,14,30]. However, prognostic and therapeutic stratification of PanNET is challenging, due to the clinical and biological heterogeneity of the disease. The current available immunohistochemical, clinical and laboratory markers are not able to identify patients for a specific therapeutic approach. Thus, characterisation of novel signalling cascades modulating tumour progression in PanNET may help to identify new prognostic and predictive markers and to overcome primary and secondary therapy resistance.

In the present study we observed a strong correlation of CUX1 expression with grading, the presence of a metastatic phenotype and worsened prognosis. These data are in line with our own previous data that identified CUX1 as driver of tumour aggressiveness in malignant insulinomas and with findings of other groups confirming a negative prognostic impact of CUX1 in various solid tumours [31,32].

The grading of PanNET is based on the proliferation capacity assessed by Ki-67 [25]. Interestingly, we were not able to demonstrate a significant relationship between Ki-67 and CUX1, possibly due to the fact that we examined samples of tissue-micro arrays (TMA) from the two cohorts, whereas reference Ki-67 was assessed by examination of the complete sections of paraffin blocks and only from primary tumours [33,34,35].

Our data identified immunohistochemical CUX1 expression as predictive marker for resistance to chemotherapy, with the mPFS being almost tripled in the CUX1 low-expressing cohort compared to the CUX1 high-expression cohort. This is in line with our recent findings that CUX1 mediates proliferation, resistance to apoptosis and angiogenesis, as demonstrated in vitro and in a xenograft model [22]. CUX1 expression was also linked to recurrence and disease-related death in human insulinomas, demonstrating a strong prognostic impact [22]. Our current study extends these findings to non-functioning PanNET indicating that CUX1 is an important mediator of progression, and serves as potential predictive biomarker for resistance to cytotoxic chemotherapy in this tumour entity. These expression data derived from human tumour tissues are functionally supported by our in vitro findings. CUX1 suppressed the caspase-9 and -3 axis, well-known key effectors of the apoptosis-machinery in cancer in all examined cell lines. The caspase-9 protease initiates intrinsic or mitochondrial apoptosis and activates caspase-3, which is the main executor molecule for programmed apoptotic cell death [36,37]. Both caspases are subject to therapeutic intervention in several ongoing trials investigating caspase activating compounds to induce apoptosis of cancer cells (e.g., NCT02355535; NCT01955434).

In addition to the regulation of effector caspases, the death associated protein kinase 1 (DAPK1) was identified as target of CUX1. However, its regulation could be only in one cell line, limiting its also DAPK1, all representing key mediators of the apoptotic machinery. The death associated protein kinase 1 (DAPK1) is a serine/threonine kinase with pleiotropic functions in cellular processes such as apoptosis, autophagy and inflammation [38]. First, DAPK1 was identified as mediator of interferon-γ induced cell death and later the role as tumour suppressor became increasingly apparent [39,40,41]. Loss of DAPK1 expression via epigenetic or transcriptional regulation was associated with highly metastatic clones of lung cancer cells, whereas the restoring of DAPK1 suppressed metastatic activity [40]. In addition, advanced tumour stage and clinically aggressive phenotypes were reported in various cancer types, where DAPK1 was lost [42,43]. Therefore, the regulation of DAPK1 by CUX1 is interesting in many respects. In the RIP1Tag2 model carcinogenesis is mediated by P53 and the retinoblastoma (RB) gene [44]. However, DAPK1 is known to interact with P53 and in case of P53 mutation DAPK1 may activate the mTOR/S6K pathway and induce proliferation [45]. Thus, the given mouse model may not reflect an optimal platform for DAPK1 function studies, but on the other hand, targeting DAPK1 could be a therapeutic approach for P53-mutant malignancies. Since CUX1 has been identified as transcriptional repressor and its activity induced tumour cell survival and migration, accompanied by tumour progression and shortened survival in several tumour entities [20,21], DAPK1 may be an integral part of the oncogenic CUX1-mediated orchestra.

## 6. Conclusions

In conclusion, our findings identify CUX1 as marker of resistance to cytotoxic chemotherapy in metastatic PanNET and as prognostic marker in PanNET after surgical resection. Functional in vitro data highly support this association by identifying effectors of the apoptosis machinery, such as caspases-3 and -9 as mediators of CUX1-induced resistance to apoptosis and tumour progression. Further work is warranted to exploit the therapeutic avenue of caspase-targeting approaches in CUX1 positive PanNET.

## Supplementary Materials

The following are available online at https://www.mdpi.com/2072-6694/12/7/1957/s1, Figure S1: (A) ROC analyses of CUX1 expression in 56 insulinoma patients. Stratification to CUX1 IRS ≥ 8, AUC 0.74, sensitivity = 67%, specificity = 71%, *p* = 0.006. (B) CUX1 score in patients with no-recurrence (*n* = 44, mean: 5.9 ± 0.49 SEM) or recurrent disease (*n* = 12, mean: 8.2 ± 1.0 SEM), *p* = 0.046. (C) In 12 patients with disease recurrence CUX1 IRS was 8.2 with mRFS and mOS of 1.5 and 11 months, respectively; Figure S2: Representative immunohistochemistry of CUX1 in primary tumours and metastases. Various IRS are also given as examples; Figure S3: qRT-PCR profiler for 84 genes of cell-cycle (A), apoptosis (B) and DNA damage (C) in Bon-1 cells with CUX1 overexpression and mock control cells of CUX1 by stably transfection. mRNA levels of genes regulated by CUX1 are figured on a logarithmic scale relative to XS13. Representative genes repressed more than threefold are revealed in the blue box; Figure S4: CUX1-dependent regulation of caspase 3 and -9 and DAPK1 on protein level by Western Blot analysis in QGP1 cells (A). Two wildtype (WT) and two CUX1 overexpression clones were used. (B) CUX1 knock-down via siRNA in comparison to siControl in QGP1 cells. Actin served as internal control; Figure S5: Representative immunohistochemistries of CUX1, DAPK1 and caspase 3 in different stages (benign and malignant tumours) of the RIP1Tag2 mouse model; Table S1: immunohistochemistries of CUX1, DAPK1 and caspase 3 in different stages (benign and malignant tumours) of the RIP1Tag2 mouse model. Clinico-pathological features of patients and association to CUX1 expression; Table S2: Clinico-pathological features of patients. Abbreviations: CTx = chemotherapy, STZ = streptozocin, Dox = doxorubicin, DTIC = dacarbacine, IF*n* = interferon, SSA = somatostatin analogues, PRRT = peptide receptor radio therapy, SIRT = selective internal radiotherapy, TACE = transarterial chemoembolisation, CR = complete response, PR = partial response, SD = stable disease, PD = progressive disease; Table S3: Clinico-pathological features of patients and association to CUX1 expression; File S1:Patients’ characteristics with grading, sites of metastases and TNM classification; File S2:Raw data of the performed gene profiler for cell cycle, apoptosis and DNA damage.
