# Supplementary material for: CUX1—Transcriptional Master Regulator of Tumor Progression in Pancreatic Neuroendocrine Tumors
Source: Cancers (Basel). 2020 Jul 19;12(7):1957. doi: 10.3390/cancers12071957 (PMC7409270; doi:10.3390/cancers12071957)

**Supplementary Materials:** **CUX1—Transcriptional Master Regulator of Tumor Progression in Pancreatic Neuroendocrine Tumors**

Sebastian Krug, Julia Weissbach, Annika Blank, Aurel Perren, Johannes Haybaeck, Volker Fendrich, Anja Rinke, Thomas Mathias Gress, Jonas Rosendahl and Patrick Michl

**Table S1.** Characteristics.

|  | **No. of patients** | **CUX1** | | ***p* Value**  **(Fisher´s exact test)** |
| --- | --- | --- | --- | --- |
|  |  | **IRS < 8** | **IRS ≥ 8** |  |
| **Gender**  **Female**  **Male** | **46**  **58** | 15  22 | 31  36 | 0.68 |
| Age  <60  ≥60 | 52  52 | 16  21 | 36  31 | 0.41 |
| Grading  G1  G2/3 | 54  47 | 24  12 | 30  35 | **0.038** |
| Ki-67  <2%  ≥2%  <5%  ≥5%  <10%  ≥10% | 39  37  49  27  63  16 | 15  10  19  6  20  15 | 24  27  30  21  43  1 | 0.37  0.23  0.33 |
| T-Stadium  T1/T2  T3/T4 | 58  36 | 21  9 | 37  27 | 0.18 |
| Metastases  None  Yes | 57  47 | 24  13 | 33  34 | **0.09** |

**Table S2.** Characteristics.

|  | **No. of Patients** | **%** |
| --- | --- | --- |
| Gender | (N = 93) |  |
| Male  Female | 52 | 55.9 |
|  | 41 | 44.1 |
| Age at diagnosis in years | | |
| Median  Range | 53 |  |
|  | 25-79 |  |
| Tumor type | | |
| Functioning  Non-functioning | 26 | 28.0 |
|  | 67 | 72.0 |
| Differentiation | | |
| NET  NEC  Unknown | 82 | 88.2 |
|  | 1 | 1.1 |
|  | 10 | 10.8 |
| Grading | | |
| G1  G2  G3  Unknown | 20 | 21.5 |
|  | 56 | 60.2 |
|  | 6 | 6.5 |
|  | 11 | 11.8 |
| Sites of metastases | | |
| None  Lymph Node  Liver  Bone  Pulmonal  Peritoneal  Spleen  Intracerebral  Other  Unknown | 2 | 2.2 |
|  | 54 | 58.1 |
|  | 85 | 91.4 |
|  | 28 | 30.1 |
|  | 5 | 5.4 |
|  | 10 | 10.8 |
|  | 4 | 4.3 |
|  | 3 | 3.2 |
|  | 12 | 12.9 |
|  | 6 | 6.5 |
| Octreotide scintigraphy | | |
| Positive  Negative  Unknown | 71 | 76.3 |
|  | 11 | 11.8 |
|  | 11 | 11.8 |
| Primary tumour resection | | |
| Yes | 29 | 31.2 |
| None | 55 | 59.1 |
| Synchronic liver metastases resection | 19 | 20.4 |
| Unknown | 9 | 9.7 |
| Prior systemic therapy | | |
| SSA | 50 | 53.8 |
| IFNα | 21 | 22.6 |
| PRRT | 4 | 4.3 |
| TACE | 16 | 17.2 |
| SIRT | 2 | 2.2 |
| Eto+Platin | 7 | 7.5 |
| No. prior systemic therapy | | |
| 0 | 17 | 18.3 |
| 1 | 29 | 31.2 |
| 2 | 26 | 28.0 |
| 3 | 6 | 6.5 |
| 4 | 1 | 1.1 |
| Unknown | 6 | 6.5 |
| Chemotherapy | | |
| STZ/Dox | 24 | 20.9 |
| STZ/5-FU | 27 | 23.5 |
| STZ/Dox/5-FU | 11 | 9.6 |
| DTIC | 28 | 24.3 |
| Both CTx regimes | 25 | 21.7 |
| Time to CTx in months | | |
| Median | 38 |  |
| Range | 1-184 |  |
| Age at CTx in years | | |
| Median | 55,5 |  |
| Range | 27-79 |  |
| Best response RECIST | |  |
| CR | 2 | 1.7 |
| PR | 34 | 28.8 |
| SD | 41 | 34.7 |
| PD | 32 | 27.1 |
| Death | | |
| Yes | 59 | 63.4 |
| No | 14 | 15.1 |
| Unknown - loss to follow up | 20 | 21.5 |

**Table S3.** Characteristics.

|  | **No. of Patients** | **CUX1** | | ***p* Value**  **(Fisher´s exact test)** |
| --- | --- | --- | --- | --- |
|  |  | **IRS < 8** | **IRS ≥ 8** |  |
| **Gender**  **Female**  **Male** | **15**  **13** | 6  9 | 9  4 | 0.15 |
| Age  <60  ≥60 | 20  8 | 9  6 | 11  2 | 0.22 |
| Diagnosis  FNA  FA | 21  6 | 10  4 | 11  2 | 0.65 |
| Grading  G1  G2/3 | 2  26 | 1  14 | 1  12 | 1.0 |
| Ki-67  <10%  ≥10% | 11  17 | 6  8 | 5  9 | 1.0 |
| SMS  Negative  Positive | 6  22 | 3  12 | 3  10 | 1.0 |
| Metastases  LK+Liver  +Other | 14  14 | 11  4 | 3  10 | **0.021** |
| Primary tumour OP  No  Yes | 12  15 | 6  9 | 6  6 | 0.71 |
| Metastases OP  No  Yes | 15  12 | 6  9 | 9  3 | 0.12 |


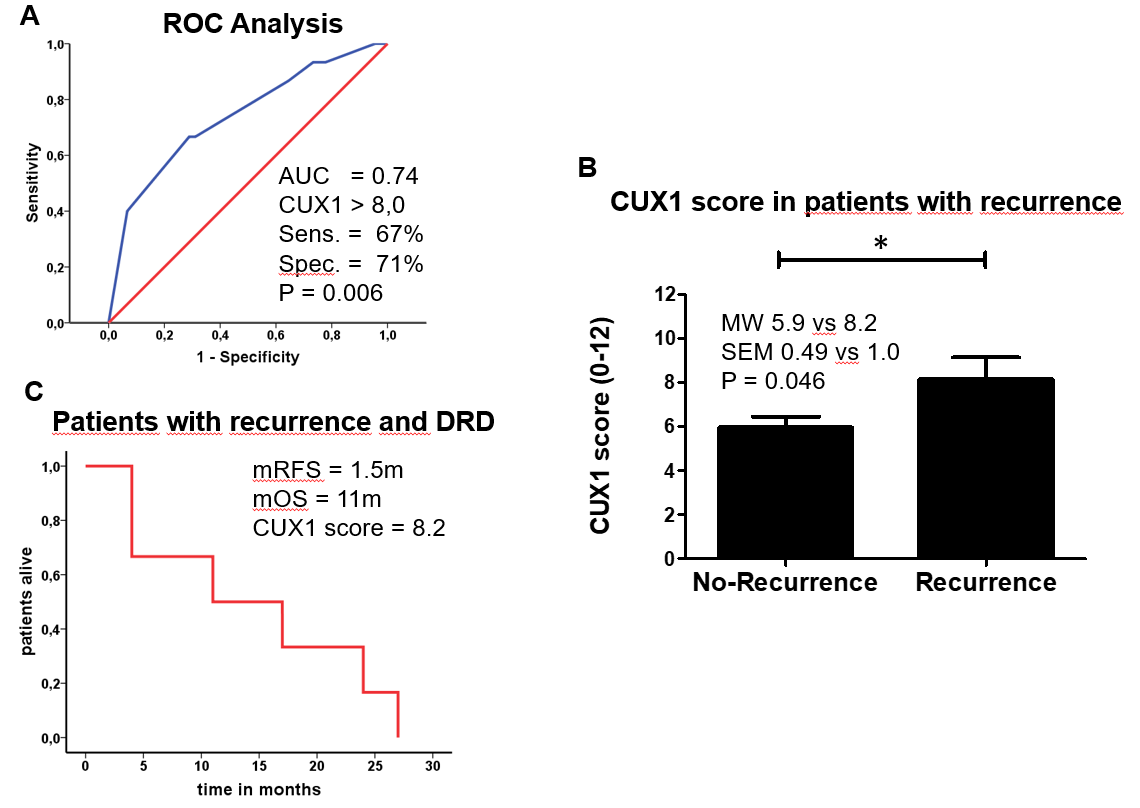


**Figure S1.** (**A**) ROC analyses of CUX1 expression in 56 insulinoma patients. Stratification to CUX1 IRS ≥ 8, AUC 0.74, sensitivity = 67%, specificity = 71%, *p* = 0.006. (**B**) CUX1 score in patients with no-recurrence (*N* = 44, mean: 5.9 ± 0.49 SEM) or recurrent disease (*N* = 12, mean: 8.2 ± 1.0 SEM), *p* = 0.046. (**C**) In 12 patients with disease recurrence CUX1 IRS was 8.2 with mRFS and mOS of 1.5 and 11 months, respectively.


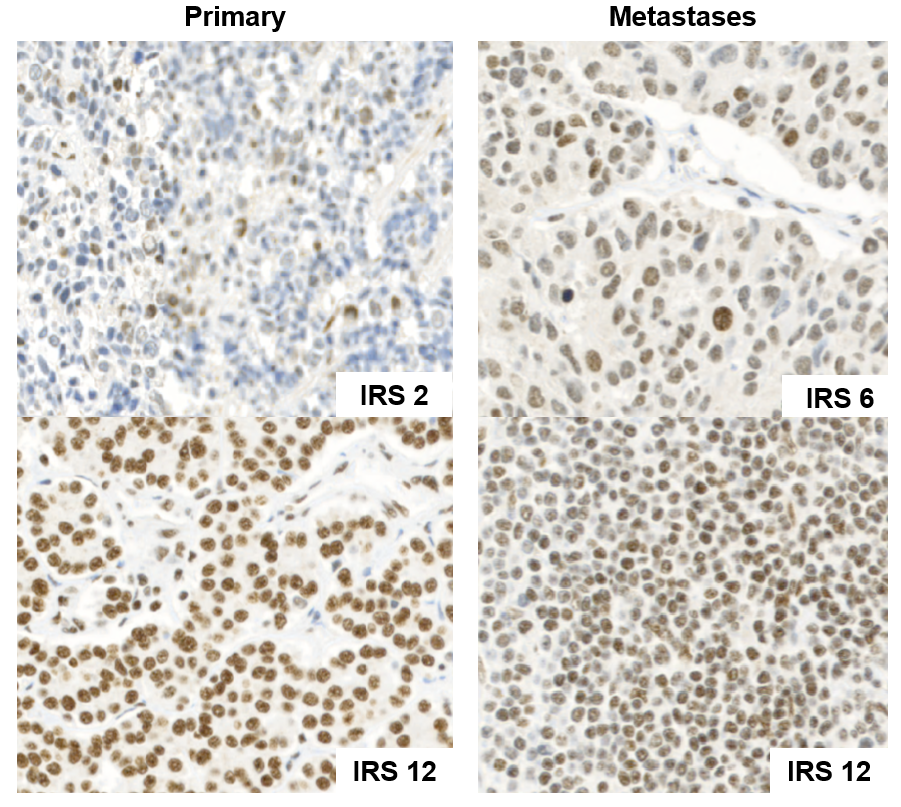


**Figure S2.** Representative immunohistochemistry of CUX1 in primary tumours and metastases. Various IRS are also given as examples.


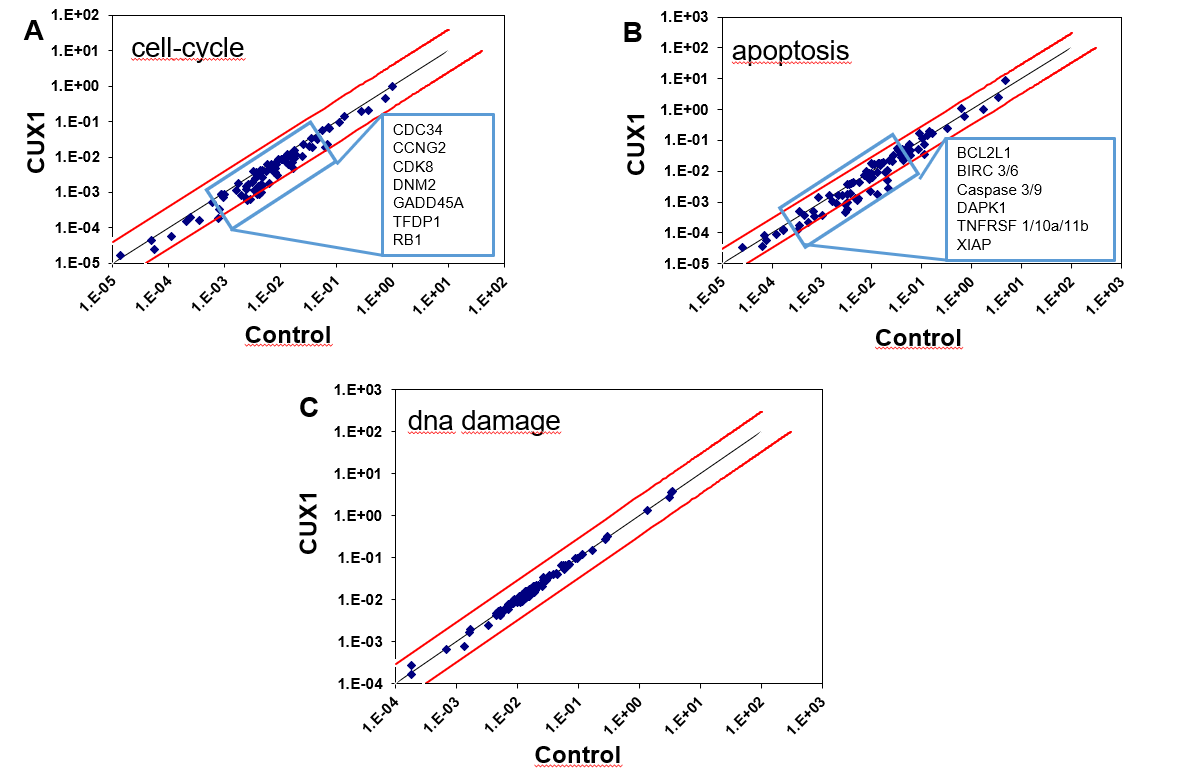


**Figure S3.** qRT-PCR profiler for 84 genes of cell-cycle (A), apoptosis (B) and DNA damage (C) in Bon-1 cells with CUX1 overexpression and mock control cells of CUX1 by stably transfection. mRNA levels of genes regulated by CUX1 are figured on a logarithmic scale relative to XS13. Representative genes repressed more than threefold are revealed in the blue box.


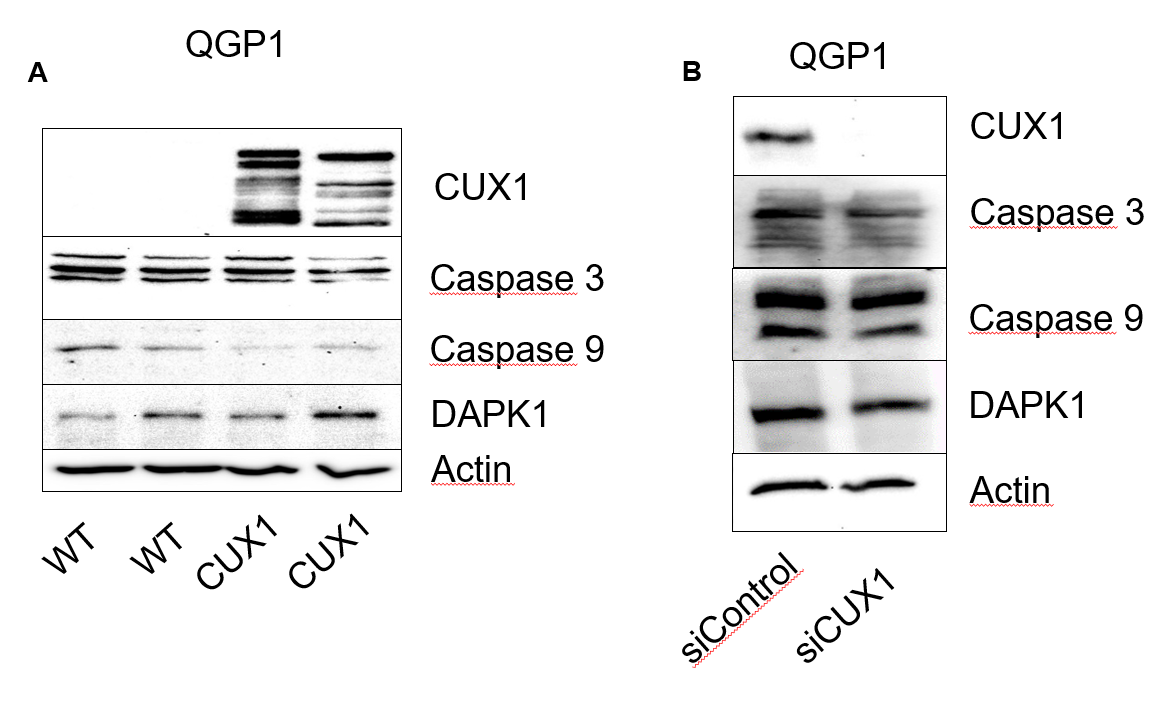


**Figure S4.** CUX1-dependent regulation of caspase 3 and -9 and DAPK1 on protein level by Western Blot analysis in QGP1 cells (A). Two wildtype (WT) and two CUX1 overexpression clones were used. (B) CUX1 knock-down via siRNA in comparison to siControl in QGP1 cells. Actin served as internal control.


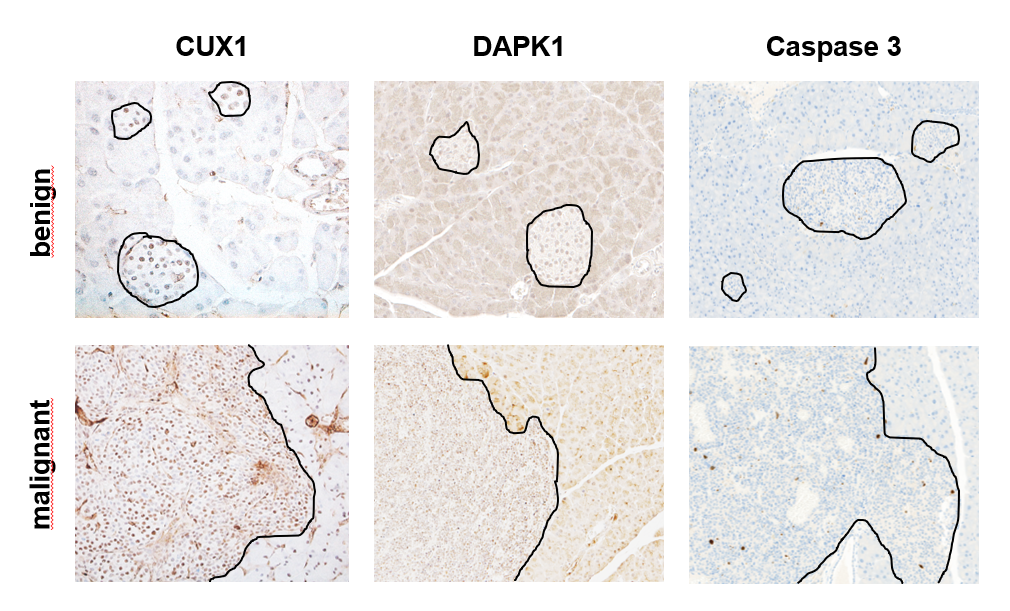


**Figure S5.** Representative immunohistochemistries of CUX1, DAPK1 and caspase 3 in different stages (benign and malignant tumours) of the RIP1Tag2 mouse model.

**The whole Western Blot of Figure 4A and 4B**


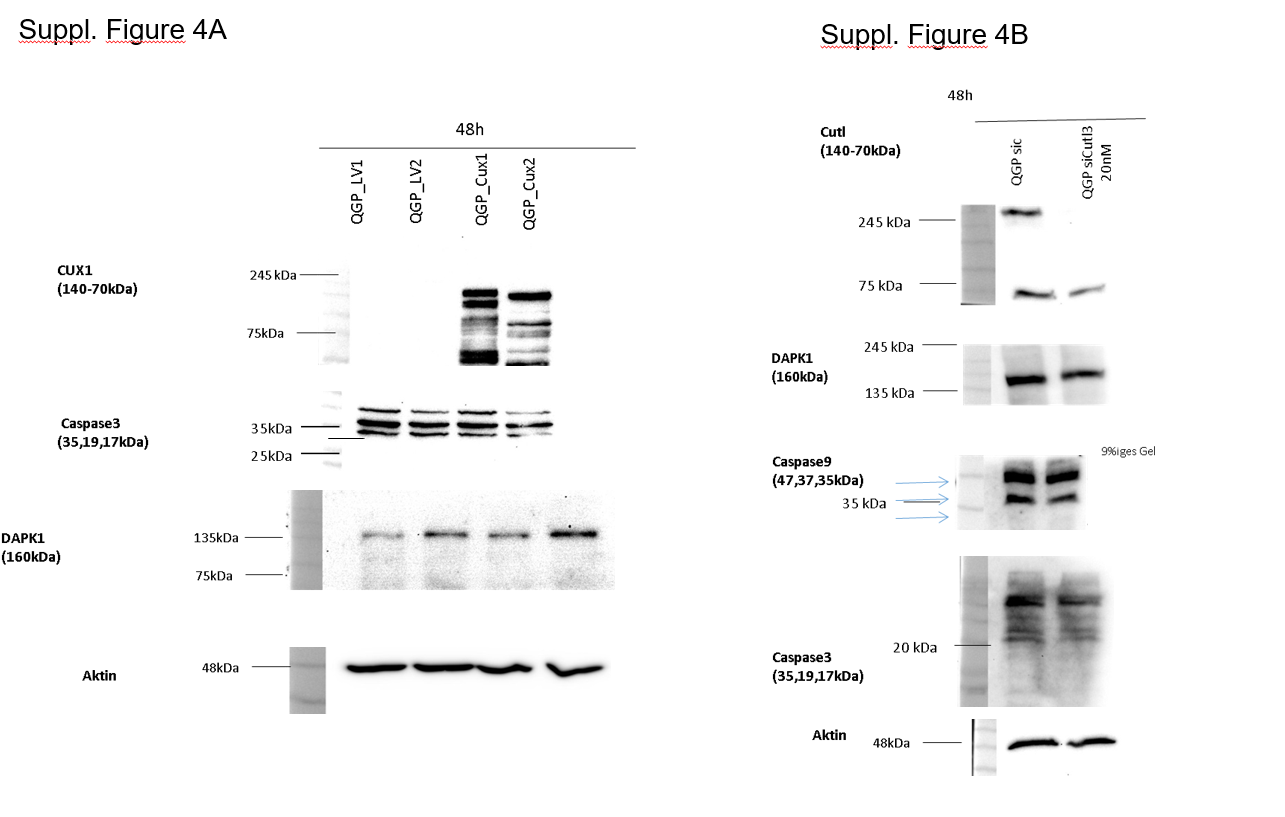

Supplement: Supplementary file 1 [file cancers-12-01957-s001.zip › cancers-837006-suppl/supplementary/cancers-837006 -Supplementary.docx]
